# Late diagnoses of Dravet syndrome: How many individuals are we missing?

**DOI:** 10.1002/epi4.12525

**Published:** 2021-08-05

**Authors:** Katri Silvennoinen, Clinda Puvirajasinghe, Kirsty Hudgell, Meneka K. Sidhu, Helena Martins Custodio, Genomics England Research Consortium, J. C. Ambrose, P. Arumugam, E. L. Baple, M. Bleda, F. Boardman‐Pretty, J. M. Boissiere, C. R. Boustred, M. J. Caulfield, G. C. Chan, C. E. H. Craig, L. C. Daugherty, A. de Burca, A. Devereau, G. Elgar, R. E. Foulger, T. Fowler, P. Furió‐Tarí, J. M. Hackett, D. Halai, A. Hamblin, S. Henderson, J. E. Holman, T. J. P. Hubbard, K. Ibáñez, R. Jackson, L. J. Jones, D. Kasperaviciute, M. Kayikci, L. Lahnstein, K. Lawson, S. E. A. Leigh, I. U. S. Leong, F. J. Lopez, F. Maleady‐Crowe, J. Mason, E. M. McDonagh, L. Moutsianas, M. Mueller, N. Murugaesu, A. C. Need, C. A. Odhams, C. Patch, D. Perez‐Gil, D. Polychronopoulos, J. Pullinger, T. Rahim, A. Rendon, P. Riesgo‐Ferreiro, T. Rogers, M. Ryten, K. Savage, K. Sawant, R. H. Scott, A. Siddiq, A. Sieghart, D. Smedley, K. R. Smith, A. Sosinsky, W. Spooner, H. E. Stevens, A. Stuckey, R. Sultana, E. R. A. Thomas, S. R. Thompson, A. Tucci, E. Walsh, S. A. Watters, M. J. Welland, E. Williams, K. Witkowska, Wendy D. Jones, Simona Balestrini, Sanjay M. Sisodiya

**Affiliations:** ^1^ Department of Clinical and Experimental Epilepsy UCL Queen Square Institute of Neurology London UK; ^2^ Chalfont Centre for Epilepsy Chalfont St Peter UK; ^3^ North East Thames Regional Genetics Service Great Ormond Street Hospital London UK; ^4^ St. Elizabeth's Centre Much Hadham Herts UK; ^5^ Genomics England London UK; ^6^ Children's Hospital A. Meyer University of Florence Florence Italy

**Keywords:** epilepsy, genetics, seizures, whole‐genome sequencing

## Abstract

We report new genetic diagnoses of Dravet syndrome in a group of adults with complex epilepsy of unknown cause, under follow‐up at a tertiary epilepsy center. Individuals with epilepsy and other features of unknown cause from our unit underwent whole‐genome sequencing through the 100 000 Genomes Project. Virtual gene panels were applied to frequency‐filtered variants based on phenotype summary. Of 1078 individuals recruited, 8 (0.74%) were identified to have a pathogenic or likely pathogenic variant in *SCN1A*. Variant types were as follows: nonsense (stopgain) in five (62.5%) and missense in three (37.5%). Detailed review of childhood history confirmed a phenotype compatible with Dravet syndrome. Median age at genetic diagnosis was 44.5 years (range 28‐52 years). Tonic‐clonic seizures were ongoing in all despite polytherapy including valproate. All had a history of fever sensitivity and myoclonic seizures, which were ongoing in two (25%) and three (37.5%) individuals, respectively. Salient features of Dravet syndrome may be less apparent in adulthood, making clinical diagnosis difficult. Regardless of age, benefits of a genetic diagnosis include access to syndrome‐specific treatment options, avoidance of harmful drugs, and monitoring for common complications.

## 1 INTRODUCTION

Dravet syndrome (DS) is one of the commonest, best‐characterized, severe, monogenic epilepsies. Individuals with DS typically present within the first year of life with convulsive seizures, often precipitated by pyrexia.^1^ Seizures become recurrent and later often include myoclonic and atypical absence seizures. Focal‐onset seizures of various semiologies are also common.^1^ Developmental delay becomes evident typically from the second year onwards.^1^ The majority of individuals have moderate to severe intellectual disability by adulthood.^2^, ^3^

Dravet syndrome is typically caused by loss of function variants in the gene *SCN1A,* particularly affecting inhibitory interneurons.^4^ The majority of pathogenic variants arise de novo.^5^ DS is now widely recognized by pediatricians and neurologists, and *SCN1A* molecular testing is available in many countries. However, older patients especially may remain undiagnosed^6^; the prevalence in age epochs across adulthood is unknown. We describe a series of individuals diagnosed with DS in adulthood based on whole‐genome sequencing.

## 2 METHODS

This study was approved by the Camden & Kings Cross Research Ethics Committee (reference 11/LO/2016). The participants did not have capacity to provide informed consent; written assent for participation was obtained from a personal consultee for each individual following the approved protocol.

Participants fulfilling criteria for the “epilepsy plus other features” category (epilepsy with structural abnormality of the brain or other organs, cognitive impairment, autism or consanguinity),^7^ with no known genetic diagnosis, were recruited to the UK 100 000 Genomes Project and underwent whole‐genome sequencing.^8^ Reads were aligned to build GRCh38 of the human genome. Virtual gene panels^9^ were chosen based on the phenotype summary entered at time of recruitment and applied to frequency‐filtered variants (Table S1). Results were reviewed in a multidisciplinary meeting with epileptology, clinical, and molecular genetics input, and classified according to the Association for Clinical Genomic Science guidelines.^10^ Further clinical data were obtained from medical records and epilepsy genomics clinic reviews.

Prior to this analysis, one individual was identified through screening within the Genomics England Research Environment for stopgain variants in the *SCN1A* gene region (chr2:165989160‐166128013).^6^ The finding was confirmed in the present analysis.

Descriptive statistics were calculated using Microsoft Excel version 16.38. Due to small sample size, central tendency was expressed using medians.

## 3 RESULTS

A total of 1078 individuals were recruited from our unit. Eight individuals (six females and two male) were found to have heterozygous pathogenic variants in *SCN1A* (Table 1). The median age at genetic diagnosis was 44.5 years (range 28‐52; Table 2). In one of the individuals (12.5%), a diagnosis of Dravet syndrome had been previously suspected by the treating physician. In three others (37.5%), electronic patient records were, in retrospect, sufficient for suspecting the diagnosis. In the remaining four (50%), sufficient details to make a clinical diagnosis of DS were not present in available electronic patient records, but subsequent review of historical (paper) notes highlighted that their phenotype was indeed compatible with DS (Table 2). All variants were absent from The Genome Aggregation Database (gnomAD).^11^ Four of the variants had been previously reported in individuals with DS, with additional functional evidence for two of these variants (Table S2). Due to the age of our patients and inability to obtain parental samples in many cases, parental testing was possible only in one individual, with confirmation of de novo status of the *SCN1A* variant.

**TABLE 1:** *SCN1A* variant details and current presentation

ID	*SCN1A* variant (all heterozygous)	Variant type; ACGS classification^10^	Age	Clinical diagnoses prior to genetic testing	Current seizures	Current ASMs	Current mobility	Current language skills	Comorbidities
1	NM_001165963.1: c.1489del: p. Arg497GlufsTer47 Prev. reported^a^	Nonsense Class 5 (PM2; PVS1)	46	1. Cryptogenic epilepsy 2. Learning disability 3. Spastic quadriplegia	TCS (2/y), MJ	OXC 1350, LEV 2000, VPA 1500, CLB 10	Crouch gait, wheelchair for longer distances	Syllables	Scoliosis Possible swallowing problems
2	NM_001165963.1: c.1754dup: p. Ser586IlefsTer2	Nonsense Class 5 (PM2; PVS1; PM6_sup)	28	1. Epilepsy with generalized tonic‐clonic seizures and episodes with eyelid fluttering. 2. Severe developmental delay of unknown etiology.	TCS (51/y); ?eyelid fluttering	PER 4, VPA 1000, LEV 250, CLB 20	Walks with crouch gait	Nonverbal	History of aspiration pneumonia
3	NM_001165963.1: c.3796G>T: p. Glu1266Ter	Nonsense Class 5 (PM2, PVS1;)	52	1. Generalized tonic‐clonic seizures 2. Spastic quadraparesis 3. Severe learning disability	TCS (7/y)	VPA 1000, CLZ 2, LEV 3000, LCM 350	Able to take walk short distances indoors; wheelchair	Nonverbal	Nil
4	NM_001165963.1: c.4003G>A: p. Val1335Met Prev. reported^a^	Missense Class 4 (PM2; PP2; PP3; PS4_mod)	51	1. Pharmacoresistant epilepsy. 2. Severe learning disability	TCS (12/y), MJ (preceding TCS)	VPA 1400, CLB 25, Primidone 625	Able to walk but unsteady	Single words	Nil
5	NM_001165963.1: c.1647C>A: p. Tyr549Ter	Nonsense Class 5 (PM2; PVS1)	34	1. Pharmacoresistant focal epilepsy 2. Learning disability	TCS (72/y)	LEV 2000, VPA 1200, LCM 300	Able to walk short distances, back hunched	Words and some phrases	Nil
6	NM_006920.4: c.664C>T: p. Arg222Ter Prev. reported^a^	Nonsense Class 5 (PM2; PVS1; PS4_mod; PM6)	43	1. Refractory partial epilepsy 2. Possible history of Landau‐Kleffner syndrome 3. Severe learning disability	TCS (1‐2/y) FIAS (30/y)	CLB 20, VPA 1100, ZON 100	Able to walk	Vocabulary 30 words, some phrases	Scoliosis. Impaired swallowing, hypothyroidism
7	NM_001165963.1: c.548T>C: p. Phe183Ser	Missense Class 4 PM2; PM1; PP2; PP3; PP4	47	1. Pharmacoresistant epilepsy 2. Possible Dravet Syndrome 3. Learning disability	TCS 5/y	TPM 500, VPA 800	Able to walk but unsteady	Nonverbal	Impaired swallowing‐modified diet
8	NM_001165963.1: c.1178G>A: p. Arg393His Prev. reported^a^	Missense Class 4 (PM2; PP2; PP3; PM5; PS4_mod)	42	1. Pharmacoresistant focal epilepsy 2. Severe learning disabilities	TCS 4/y, MJ	ZON 400, VPA 1600, LCM 400	Gait ataxic and slow	Speaks in sentences, able to have basic conversation	Scoliosis

Abbreviations: ACGS, Association for Clinical Genomic Science; CBZ, carbamazepine; CLB, clobazam; FIAS, focal‐onset nonmotor seizures with impaired awareness; LCM, lacosamide; LEV, levetiracetam; LTG, lamotrigine; MJ, myoclonic jerks; OXC, oxcarbazepine; PER, perampanel; TCS, tonic‐clonic seizures; VAP, valproate; ZON, zonisamide.

^a^ Details of previous reports may be found in Table S2.

**TABLE 2:** History of epilepsy and development

ID	Gender	Age and type of seizure onset	Age at onset of developmental delay	Motor functions/mobility at best	Language abilities at best	Overall cognitive abilities at best as assessed by psychology	Behavioral/neuropsychiatric history	First record of abnormal EEG	Seizure types ever	N^o^ of previous ASM/therapies	Response to SCBs
1	M	2.5 mo; febrile focal motor	15 mo	Age 4: “Loved climbing and escaping”; walked independently without problems	Single words/2‐word phrases; 1‐2‐ yo level (age 29)	2‐3 yo level (age 29)	Hyperactive, occasional aggressive behavior, difficulties getting out of car. Low mood as adult	Age 15 mo (details unknown)	TCS, MJ, FIAS, “head nodding and arms outstretched”	11	LTG—deterioration
2	F	6 mo; febrile TCS Possibly episodes of eye deviation from age 4 mo	3 y	Able to run	Single words	N/A	N/A	Age 12 y—encephalopathic	TCS, MJ, eyelid fluttering with cessation of activity	8	N/A
3	M	9 mo; febrile	4 y	Able to run, cycle, play ball games (around age 8‐10)	Vocabulary 70 words; short phrases (age 21 y)	N/A	Hyperactive; can spend hours on some activities	Age 10 y—encephalopathic	TCS, MJ, dialeptic, focal motor onset	12	LTG—deterioration in MJ LCM helpful
4	F	6 mo; febrile 6 h after vaccination	2‐3 y	Age 17—enjoys indoor hockey, good at throwing, and catching balls	Short 2‐3 word phrases, many single word utterances—at level of 2.7 yo (age 18 y)	N/A	Compulsive, hyperactive by age 5 “Can be very obsessive when it comes to use of free time”	Age 1.5 y—encephalopathic	TCS, myoclonic jerks, “drop attacks”	5	CBZ—deterioration
5	F	10 mo; nonfebrile	3 y	Able to run	Words and some phrases	N/A	N/A	Age 9.5 y—encephalopathic, ill‐formed discharges	TCS, MJ preceding TCS	15	N/A
6	F	4 mo; febrile TCS	2 y	Poor balance but able to run	Words and some phrases	2 yo level (age 8 y)	Age 2—hyperactive, short attention span Phobias and anxiety as adult	Age 8 y—encephalopathic	TCS, MJ, FIAS	10	N/A
7	F	6 mo; febrile; within 24 h of vaccination	N/A	N/A	N/A	N/A	Manic episode as adult	Second EEG within 1st year of life—abnormal	TCS, MJ, FIAS	9	Deterioration on LTG
8	F	9 mo; febrile hemiclonic	3 y	Able to ride a bike, hop, and jump, climb (age 6.5), also roller‐skate	Speaks in sentences, able to have basic conversation	4‐5yo level (age 15 y)	Hyperactive from age 3 y	Age 2 y—excess of slow	TCS, MJ, FIAS, tonic, focal motor onset	12	LTG—longer recovery & duration of seizures

Abbreviations: CBZ, carbamazepine; FIAS, focal‐onset nonmotor seizures with impaired awareness; LTG, lamotrigine; MJ, myoclonic jerks; SCBs, sodium channel blocker; TCS, tonic‐clonic seizures.

None of these individuals had any additional filtered variants felt to be contributing to their phenotype.

The median age of seizure onset was 6 months (2.5‐10). In seven (87.5%), the first seizure occurred in the context of pyrexia. Two individuals had received a vaccination in the preceding 24 hours.

Median age of onset of developmental delay was 2.5 years (range 1.25‐4). All patients had a history of bilateral tonic‐clonic seizures (TCS) and myoclonic jerks. Six individuals had a history of focal‐onset nonmotor seizures with impaired awareness (FIAS); EEGs were not available to confirm atypical absences. Other seizure types ever included focal‐onset motor seizures (two individuals), unclassified drops/episodes of head nodding (two individuals), and tonic seizures (one individual). All patients had ongoing TCS. Myoclonic seizures were ongoing in three. FIAS continued in one. One patient had unclassified episodes of eyelid fluttering. Fever or intercurrent illness was elicited as an ongoing seizure precipitant in two.

Seven (87.5%) patients had data on previous and current motor and language skills. All seven had deterioration in mobility compared to their best‐attained level; however, all continued to be able to walk for at least short distances. Language skills ranged from no verbal communication to ability to have a basic conversation using sentences. Four of seven (57.1%) had deterioration in language skills compared to their best level.

All patients were taking valproate at current presentation. The median number of current anti‐seizure treatments (including ketogenic diet) was 3 (range 2‐4). The median number of previously tried anti‐seizure treatments (excluding rescue medications) was 11 (range 5‐15). All patients had a history of sodium channel blocker (SCB) treatment. Five individuals (62.5%) had documented deterioration in seizure frequency and/or severity while on lamotrigine or carbamazepine.

## 4 DISCUSSION

Dravet syndrome is among the most common monogenic epileptic encephalopathies, with an estimated population‐based incidence of about 1/15500 live births.^12^ Although some individuals succumb in childhood, recent estimates suggest over 80% will require care in adult services.^13^ We conclude, therefore, that a number of adult patients are currently undiagnosed and have unmet health needs. Our experience highlights the need to consider a genetic diagnosis among older individuals with treatment‐resistant epilepsy.

Dravet syndrome is now, and has been historically, typically diagnosed in childhood; therefore, the commonly appreciated key clinical features reflect the childhood presentation. It is recognized that TCS persist in adulthood in the majority of individuals with DS, while seizure types characteristic in childhood, including myoclonic seizures and atypical absence seizures, continue to occur only in a minority.^2^, ^3^, ^14^ In our series also, TCS were ongoing in all, while half of patients had no other definite seizures. In previous series of adult with DS, gait impairment of variable severity, including crouch gait, and significant language impairment were reported in the majority^2^, ^3^; swallowing difficulties are also a recognized late feature in some.^3^ In keeping with the previous literature, all our patients had at least one of these three features. While nonspecific, these features might alert to a possible diagnosis of DS in adults with refractory epilepsy.

In our group of adults with epilepsy and other features, a new genetic diagnosis of DS could be made in 0.74%, a relatively high proportion for a single syndrome. In our view, all adults with refractory epilepsy and intellectual disability of unknown cause should be suspected of having a possible genetic cause, including DS, and be offered genetic testing. In those with seizure onset before age of 1 year, fever sensitivity, and history of myoclonic seizures, testing for *SCN1A* variants might be undertaken directly. While reviewing the childhood notes of all adults with refractory epilepsy for features of syndromic diagnoses would seem prudent, in reality, such notes may not be available and such review would be a sizeable task in large busy clinics. Panels incorporating a number of genes associated with epilepsy, such as those used in this study (Table S1), provide a cost‐effective way to screen for variants in multiple genes, including other genes associated with a Dravet‐like phenotype.

Among the widely available anti‐seizure medications (ASMs), established treatments for DS include valproate, clobazam, and topiramate.^15^ Despite lack of a syndromic diagnosis, all our patients had arrived at polytherapy incorporating valproate and half also took regular clobazam. Despite these treatments, all continue to have TCS. Emerging or licensed treatments for DS include stiripentol, cannabidiol, and fenfluramine.^15^ Establishing a diagnosis of DS may help fulfill local criteria necessary for access to these drugs or future treatments on a research basis or through early access programs.

One of the diagnostic clues for DS is exacerbation of seizures by SCBs,^15^ and avoiding these presents one of the earliest genetics‐driven treatment approaches.^16^ All of our patients had a history of SCB use; in five, this was associated with clearlydocumented exacerbation of seizures. One of these patients remains on oxcarbazepine. Withdrawal of SCBs has been associated with benefit also in older individuals^3^ and will be considered in this patient.

A multidisciplinary approach is helpful to address the common complications of DS that include dysphagia and progressive gait problems.^3^ Making the diagnosis allows for appropriate monitoring and therapy input as necessary. People with DS are at high risk of sudden unexpected death in epilepsy (SUDEP),^17^ providing further motivation to optimize seizure control. Arriving at a genetic diagnosis may provide an end to a decades‐long diagnostic odyssey for families. The diagnosis may also have implications in terms of genetic counseling for the wider family, as some causal variants may be inherited.

Estimation of rare disease prevalence is a step forward in promoting disease‐specific treatments, as prevalence influences funding priorities and is helpful for planning of clinical trials. Estimation of the prevalence of DS in adulthood currently relies on incidence at birth.^12^, ^13^ We suggest our cross‐sectional study highlights the need for widespread access to genetic testing among adults with treatment‐resistant epilepsies, as there are clearly undiagnosed adults.

## CONFLICT OF INTEREST

None of the authors has any conflict of interest to disclose. We confirm that we have read the Journal's position on issues involved in ethical publication and affirm that this report is consistent with those guidelines.

## Supporting information

Table S1‐S2

## Data Availability

Access to genetics data of the 100 000 Genomes Project may be obtained via membership of the Genomics England Clinical Interpretation Partnership (GeCIP; https://www.genomicsengland.co.uk/about‐gecip/joining‐research‐community/).

## APPENDIX 1

Genomics England Research Consortium contributors: J. C. Ambrose^5^, P. Arumugam^5^, E. L. Baple^5^, M. Bleda^5^, F. Boardman‐Pretty^5,7^, J. M. Boissiere^5^, C. R. Boustred^5^, M. J. Caulfield^5,7^, G. C. Chan^5^, C. E. H. Craig^5^, L. C. Daugherty^5^, A. de Burca^5^, A. Devereau^5^, G. Elgar^5,7^, R. E. Foulger^5^, T. Fowler^5^, P. Furió‐Tarí^5^, J. M. Hackett^5^, D. Halai^5^, A. Hamblin^5^, S. Henderson^5,7^, J. E. Holman^5^, T. J. P. Hubbard^5^, K. Ibáñez^5,7^, R. Jackson^5^, L. J. Jones^5,7^, D. Kasperaviciute^5,7^, M. Kayikci^5^, L. Lahnstein^5^, K. Lawson^5^, S. E. A. Leigh^5^, I. U. S. Leong^5^, F. J. Lopez^5^, F. Maleady‐Crowe^5^, J. Mason^5^, E. M. McDonagh^5,7^, L. Moutsianas^5,7^, M. Mueller^5,7^, N. Murugaesu^5^, A. C. Need^5,7^, C. A. Odhams^5^, C. Patch^5,7^, D. Perez‐Gil^5^, D. Polychronopoulos^5^, J. Pullinger^5^, T. Rahim^5^, A. Rendon^5^, P. Riesgo‐Ferreiro^5^, T. Rogers^5^, M. Ryten^5^, K. Savage^5^, K. Sawant^5^, R. H. Scott^5^, A. Siddiq^5^, A. Sieghart^5^, D. Smedley^5,7^, K. R. Smith^5,7^, A. Sosinsky^5,7^, W. Spooner^5^, H. E. Stevens^5^, A. Stuckey^5^, R. Sultana^5^, E. R. A. Thomas^5,7^, S. R. Thompson^5^, A. Tucci^5,7^, E. Walsh^5^, S. A. Watters^5^, M. J. Welland^5^, E. Williams^5^, K. Witkowska^5,7^.

^5^Genomics England, London, UK.

^7^William Harvey Research Institute, Queen Mary University of London, London, EC1M 6BQ, UK.
