# Supplementary material for: Late diagnoses of Dravet syndrome: How many individuals are we missing?
Source: Epilepsia Open. 2021 Aug 5;6(4):770–6. doi: 10.1002/epi4.12525 (PMC8633473; doi:10.1002/epi4.12525)
Supplement: Supplementary file 1 — Table S1‐S2 [file EPI4-6-770-s001.docx]

**Supporting information to**

**Late diagnoses of Dravet syndrome: How many individuals are we missing?**

Katri Silvennoinen, Clinda Puvirajasinghe, Kirsty Hudgell, Meneka K, Sidhu, Helena Martins Custodio, Genomics England Research Consortium, Wendy D. Jones, Simona Balestrini, Sanjay M. Sisodiya

**Table S1. Genomics England PanelApp (1) virtual gene panels applied in the genome-interpretation pipeline for each participant.**

| ID | Panels |
| --- | --- |
| 1 | Intellectual disability v2.50; Undiagnosed metabolic disorders v1.73; Epileptic encephalopathy v1.132; Mitochondrial disorders v1.66 |
| 2 | Epileptic encephalopathy v1.132; Intellectual disability v2.50; Mitochondrial disorders v1.66; Undiagnosed metabolic disorders v1.73 |
| 3 | Undiagnosed metabolic disorders v1.77; Mitochondrial disorders v1.66; Intellectual disability v2.393; Epileptic encephalopathy v1.143 |
| 4 | Mitochondrial disorders v1.153; Intellectual disability v2.800; Undiagnosed metabolic disorders v1.104; Genetic epilepsy syndromes v1.35 |
| 5 | Intellectual disability v2.800; Undiagnosed metabolic disorders v1.105; Mitochondrial disorders v1.291;Genetic epilepsy syndromes v1.35 |
| 6 | Intellectual disability v2.800; Undiagnosed metabolic disorders v1.105; Mitochondrial disorders v1.291; Genetic epilepsy syndromes v1.35 |
| 7 | Intellectual disability v2.833; Undiagnosed metabolic disorders v1.105; Mitochondrial disorders v1.292; Genetic epilepsy syndromes v1.38 |
| 8 | Intellectual disability v2.1046; Genetic epilepsy syndromes v1.336 |

Details of panels may be found at https://panelapp.genomicsengland.co.uk/panels/

**Table S2: Details of previous reports of the *SCN1A* variants identified in people in this study, where applicable.**

| **Variant details** | **Clinical databases and publications** | | |
| --- | --- | --- | --- |
|  | **Published cases*** | **Functional evidence** | **If no publications, reports of individuals in ClinVar** |
| NM_001165963.1:c.1489del;  p.Arg497GlufsTer47 | None | None | Single SMEI case |
| NM_001165963.1:c.1754dup;  p.Ser586IlefsTer2 | None | None | None |
| NM_001165963.1:c.3796G>T;  p.Glu1266Ter | None | None | None |
| NM_001165963.1:c.4003G>A;  p.Val1335Met | SMEI(2,3) | None | Not applicable |
| NM_001165963.1:c.1647C>A  NP_001159435.1:p.Tyr549Ter | None | None | None |
| NM_006920.4:c.664C>T;  p.Arg222Ter | SMEI/DS (4–15)(7)  “SMEI–GEFS+” (16)  “Borderline SMEI” (17)  DEE (18,19)  “Intractable childhood epilepsy” (11) | Absent sodium current (20) | Not applicable |
| NM_001165963.1:c.548T>C;  p.Phe183Ser | None | None | None |
| NM_001165963.1:c.1178G>A;  p.Arg393His | SMEI/DS (10,12,13,15,21–27)  “Intractable childhood epilepsy” (11) | Absent sodium current(28) | Not applicable |

Abbreviations: DEE – developmental and epileptic encephalopathy; DS – Dravet syndrome; GEFS+ – genetic epilepsy with febrile seizures plus; SMEI – severe myoclonic epilepsy of infancy

* identified from ClinVar and <http://scn1a.caae.org.cn/>

**Supplementary References**

1. Martin AR, Williams E, Foulger RE, Leigh S, Daugherty LC, Niblock O, et al. PanelApp crowdsources expert knowledge to establish consensus diagnostic gene panels. Nature Genetics 2019;51:1560–1565.

2. Zucca C, Redaelli F, Epifanio R, Zanotta N, Romeo A, Lodi M, et al. Cryptogenic epileptic syndromes related to SCN1A: Twelve novel mutations identified. Arch Neurol 2008;65:489–94.

3. Sun H, Zhang Y, Liang J, Liu X, Ma X, Qin J, et al. Seven novel SCN1A mutations in Chinese patients with severe myoclonic epilepsy of infancy. Epilepsia 2008;49; 1104–7.

4. Claes L, Del-Favero J, Ceulemans B, Lagae L, Van Broeckhoven C, De Jonghe P. De novo mutations in the sodium-channel gene SCN1A cause severe myoclonic epilepsy of infancy. Am J Hum Genet. 2001;68:1327–32.

5. Nabbout R, Gennaro E, Dalla Bernardina B, Dulac O, Madia F, Bertini E, et al. Spectrum of SCN1A mutations in severe myoclonic epilepsy of infancy. Neurology 2003;60:1961–7.

6. Lee HF, Chi CS, Tsai CR, Chen CH, Wang CC. Electroencephalographic features of patients with SCN1A-positive dravet syndrome. Brain Dev 2015;37:599–611.

7. Esterhuizen AI, Mefford HC, Ramesar RS, Wang S, Carvill GL, Wilmshurst JM. Dravet syndrome in South African infants: Tools for an early diagnosis. Seizure 2018;62:99–105.

8. Fukuma G, Oguni H, Shirasaka Y, Watanabe K, Miyajima T, Yasumoto S, et al. Mutations of Neuronal Voltage-gated Na+ Channel α1 Subunit Gene SCN1A in Core Severe Myoclonic Epilepsy in Infancy (SMEI) and in Borderline SMEI (SMEB). Epilepsia 2004;45:140–8.

9. Vadlamudi L, Dibbens LM, Lawrence KM, Iona X, McMahon JM, Murrell W, et al. Timing of De Novo Mutagenesis — A Twin Study of Sodium-Channel Mutations. N Engl J Med 2010;363:1335–40.

10. Lemke JR, Riesch E, Scheurenbrand T, Schubach M, Wilhelm C, Steiner I, et al. Targeted next generation sequencing as a diagnostic tool in epileptic disorders. Epilepsia 2012;53:1387–98.

11. Wang JW, Shi XY, Kurahashi H, Hwang SK, Ishii A, Higurashi N, et al. Prevalence of SCN1A mutations in children with suspected Dravet syndrome and intractable childhood epilepsy. Epilepsy Res 2012;102:195–200.

12. Xu X, Zhang Y, Sun H, Liu X, Yang X, Xiong H, et al. Early clinical features and diagnosis of Dravet syndrome in 138 Chinese patients with SCN1A mutations. Brain Dev. 2014;36:676–81.

13. Margherita Mancardi M, Scapolan S, dalla Bernardina B, Bertini E, Bianchi A, Romeo A, et al. Familial Occurrence of Febrile Seizures and Epilepsy in Severe Myoclonic Epilepsy of Infancy (SMEI) Patients with SCN1A Mutations. Epilepsia 2006;47:1629–35.

14. Depienne C, Trouillard O, Saint-Martin C, Gourfinkel-An I, Bouteiller D, Carpentier W, et al. Spectrum of SCN1A gene mutations associated with Dravet syndrome: Analysis of 333 patients. J Med Genet. 2009;46:183–91.

15. Zuberi SM, Brunklaus A, Birch R, Reavey E, Duncan J, Forbes GH. Genotype-phenotype associations in SCN1A-related epilepsies. Neurology 2011;76:594–600.

16. Orrico A, Galli L, Grosso S, Buoni S, Pianigiani R, Balestri P, et al. Mutational analysis of the SCN1A, SCN1B and GABRG2 genes in 150 Italian patients with idiopathic childhood epilepsies. Clin Genet. 2009;75:579–81.

17. Harkin LA, McMahon JM, Iona X, Dibbens L, Pelekanos JT, Zuberi SM, et al. The spectrum of SCN1A-related infantile epileptic encephalopathies. Brain 2007;130:843–52.

18. Hamdan FF, Myers CT, Cossette P, Lemay P, Spiegelman D, Laporte AD, et al. High Rate of Recurrent De Novo Mutations in Developmental and Epileptic Encephalopathies. Am J Hum Genet 2017;101:664–85.

19. Lindy AS, Stosser MB, Butler E, Downtain-Pickersgill C, Shanmugham A, Retterer K, et al. Diagnostic outcomes for genetic testing of 70 genes in 8565 patients with epilepsy and neurodevelopmental disorders. Epilepsia 2018;59:1062–71.

20. Bechi G, Scalmani P, Schiavon E, Rusconi R, Franceschetti S, Mantegazza M. Pure haploinsufficiency for Dravet syndrome Na V1.1 (SCN1A) sodium channel truncating mutations. Epilepsia 2012;53:87–100.

21. Le SV, Le PHT, Le TK Van, Kieu Huynh TT, Hang Do TT. A mutation in *GABRB3* associated with Dravet syndrome. Am J Med Genet Part A 2017;173:2126–31.

22. Claes L, Ceulemans B, Audenaert D, Smets K, Löfgren A, Del-Favero J, et al. De novo SCN1A mutations are a major cause of severe myoclonic epilepsy of infancy. Hum Mutat. 2003;21:615–21.

23. Marini C, Mei D, Temudo T, Ferrari AR, Buti D, Dravet C, et al. Idiopathic epilepsies with seizures precipitated by fever and SCN1A abnormalities. Epilepsia. 2007;48:1678–85.

24. Sun H, Zhang Y, Liu X, Ma X, Yang Z, Qin J, et al. Analysis of SCN1A mutation and parental origin in patients with Dravet syndrome. J Hum Genet. 2010;55:421–7.

25. Lim BC, Hwang H, Chae JH, Choi JE, Hwang YS, Kang SH, et al. SCN1A mutational analysis in Korean patients with Dravet syndrome. Seizure. 2011;20:789–94.

26. Rilstone JJ, Coelho FM, Minassian BA, Andrade DM. Dravet syndrome: Seizure control and gait in adults with different SCN1A mutations. Epilepsia. 2012;53:1421–8.

27. Allen AS, Berkovic SF, Cossette P, Delanty N, Dlugos D, Eichler EE, et al. De novo mutations in epileptic encephalopathies. Nature. 2013;501:217–21.

28. Ohmori I, Kahlig KM, Rhodes TH, Wang DW, George AL. Nonfunctional SCN1A is common in severe myoclonic epilepsy of infancy. Epilepsia. 2006;47:1636–42.
